# Smartphone-Based Cancer and Obesity Prevention Education Program for Chinese Women (SCOPE): A Pilot RCT

**DOI:** 10.3390/ijerph20105768

**Published:** 2023-05-09

**Authors:** Jyu-Lin Chen, Jia Guo, Qinyi Zhong, Yuanyuan Jiang, Honghui Zhang, Ping Mao, Qinyuan Huang, Chen-Xi Lin, Thomas J. Hoffmann

**Affiliations:** 1 School of Nursing, University of California, San Francisco, CA 94143, USA; 2 School of Nursing, Central South University, Changsha 410017, China; 3 Manchester Centre for Health Psychology, University of Manchester, Manchester M13 9PL, UK; 4 Hunan Provincial People’s Hospital, The First-Affiliated Hospital of Hunan Normal University, Changsha 410081, China; 5 Nursing Department, The Third Xiangya Hospital, Central South University, Changsha 410013, China; 6 Department of Biostatistics and Epidemiology, University of California, San Francisco, CA 94143, USA

**Keywords:** smartphone-based, cancer prevention, obesity prevention, breast cancer, education program

## Abstract

Breast cancer prevalence has increased globally, with 12.2% of breast cancer cases identified in China. Obesity and unhealthy lifestyles are major risk factors for breast cancer. We conducted a randomized control trial to assess the feasibility and evaluate the preliminary effect of the Smartphone-Based Cancer and Obesity Prevention Education (SCOPE) program among adult biological women with a waist circumference greater than 80 cm. The SCOPE program includes tailored and culturally appropriate educational information for obesity and breast cancer prevention delivered by the research team via WeChat. The control group received non-tailored general health information via WeChat. A total of 102 women (52 intervention, 50 control) participated, and 87 (85%) completed 6-month follow-up assessments. For the primary study outcome at 6 months, women using SCOPE significantly reduced waist circumference (Cohen’s *d* = −0.39, *p* < 0.001). For secondary outcomes at 6 months, women using SCOPE significantly reduced BMI (d = −0.18, *p* = 0.001) and increased breast cancer-related knowledge (d = 0.48, *p* = 0.001) and attitude (d = 1.39, *p* < 0.01). No significant findings were found regarding diet self-efficacy, physical self-efficacy, or breast cancer screening barriers. The results suggest the intervention has great potential to promote the health and wellness of women.

## 1. Introduction

Cancer is China’s leading cause of death (about 7500 deaths per day). Cancer not only adversely affects the health of individuals but also imposes an economic burden on families and healthcare systems [1]. The leading causes of death in Chinese women include breast and endometrial cancer. China carries a significant burden of breast cancer, accounting for about 12.2% of newly diagnosed breast cancer globally and 9.6% of related deaths [2,3]. Rapid urbanization, lifestyle changes (e.g., unhealthy diet and physical inactivity), and obesity, especially abdominal obesity, are crucial risk factors for breast and endometrial cancers in Chinese women [2,3,4,5,6,7]. Among premenopausal women, studies have found that abdominal obesity increases breast cancer risk by 1.09-fold, and endometrial cancer risk ranges from 1.21–1.27 fold [2,6]. Mothers with dependent children are at greater risk of unhealthy lifestyle behaviors and obesity, which are often intertwined [8,9]. Therefore, it is essential to reduce obesity and improve a healthy lifestyle as they are key elements to breast cancer prevention. However, research on reducing obesity and breast cancer risk is limited, especially among women with abdominal obesity and dependent children in China. Developing interventions to reduce the risk of abdominal obesity and breast cancer in Chinese women before they reach menopause is warranted.

In China, smartphone ownership is about 71%; thus, a smartphone-based intervention could offer an effective and feasible tool to reduce the risk of obesity and breast cancer [10]. Smartphones or internet-based medical and health support, such as health and fitness apps, telemedicine, and prescription management, has been widely adopted in China and worldwide during the COVID pandemic—[11,12], where regular activities have been limited, especially during shelter-in-place orders. In early 2020 and during shelter-in-place, many cities were on lockdowns, and individuals were asked to stay home with limited ability to go out. Social media has been important in providing social support and conveying health information [13]. Studies have found that social media’s unique communication abilities enabled it to effectively share medical and health information during the COVID pandemic [14,15]. Several researchers have found that overweight and obese adults who used a smartphone app lost significantly more weight than those who did not (1.04–6.8 kg) [16,17,18,19]. However, most of these interventions used only four techniques for behavior change (instruction, goal setting, self-monitoring, and general feedback) without considering the participants’ behavior, culture, and preferences. Furthermore, none of these studies focused on premenopausal mothers with abdominal obesity. Developing effective and sustainable interventions tailored to an individual’s cultural lifestyle and improving social support are essential for obesity and cancer prevention [20,21].

Thus, we piloted a smartphone-based intervention designed to meet the needs and lifestyles of Chinese mothers using the popular communication platform WeChat. This study aimed to assess the feasibility and estimate the preliminary efficacy of a Smartphone-Based Cancer and Obesity Prevention Education Program for Chinese Women (SCOPE) using a randomized control trial (RCT) design. To prevent the risk for breast cancer, this study focused on obesity as the primary outcome and healthy lifestyle and knowledge and attitude regarding breast cancer prevention as secondary outcomes. Two major aims of this study include:

Aim 1: To assess the feasibility of SCOPE, a tailored smartphone-based lifestyle intervention.

Aim 2: To estimate the preliminary efficacy of SCOPE intervention on the primary study outcome (waist circumference) and secondary outcomes (body mass index, fruit and vegetable consumption, physical activity, self-efficacy in diet and physical activity, social support in diet and physical activity, and knowledge and attitudes toward breast cancer) in the SCOPE intervention compared to the control groups at 3 months and 6 months.

## 2. Materials and Methods

This pilot study used a randomized control trial design. The social cognitive theory (SCT) [22,23] and social determinants of health (SDH) were used as the study’s framework [24,25] because both theories acknowledge cognitive, social, environmental, and cultural influences to be important factors in behavioral change and population health. The SCT views human behavior as an interaction between a person, a behavior, and a context (environment). At the same time, SDH explains how non-medical factors (such as age, gender, race, income, education, and residency) affect health [22,23,24,25]. The SCOPE intervention aims to increase self-efficacy and decrease abdominal obesity and the risk of breast cancer by (1) setting realistic and achievable goals for targeted behavioral changes (outcome expectation), (2) providing necessary skills (skill mastery), (3) improving self-regulation in maintaining healthy behaviors and a healthy waist circumference (self-regulation capabilities), and (4) improving social support by developing a social network to reduce waist circumference, promote healthy lifestyles, increase cancer screening, and improve stress management (environmental and social factors). Because women with children tend to have less time to attend educational sessions in person, using technology, including social networking, can reduce barriers to health information and increase social support. This has been especially important during the COVID pandemic when shelter-in-place was enforced. The intervention integrates SCT, SDH, and standard technology (WeChat). It is tailored to the behavior patterns, goals, environment, and cultures of premenopausal women with abdominal obesity who have dependent children in China. The modules include information and activities they can do with and without their child(ren) and families, as well as indoor and outdoor options.

Women who met the inclusion criteria from two community health centers in Changsha, Hunan Province, China, were invited to this study and randomized to either the SCOPE or control programs. Inclusion criteria included (1) being biologically female, (2) being at least 18 years old, (3) having a waist circumference greater than 80 cm, (4) owning a smartphone, (5) being able to read Chinese and speak Mandarin, (6) being premenopausal, and (7) having a child between the ages of 1 and 17 years old. Exclusion criteria included (1) being pregnant, (2) giving birth less than 12 months before the enrollment date, or (3) having an acute or life-threatening disease (e.g., renal or heart failure).

Trained research assistants worked with the study sites to recruit eligible women. An invitation letter that explains the study’s purpose and WeChat contact information was sent to all women with dependent children. They were asked to message the study’s WeChat within two weeks of receiving the invitation to indicate their interest in participating. Those who were interested were invited to an in-person screening session during health fairs hosted at the recruitment sites. Several in-person screening sessions were offered at each location. During these sessions, eligibility verification, informed consent, administration of a baseline survey, and measurements were obtained. The committee on human research at the University of California San Francisco and Central South University approved the study (IRB 18-27025).

The SCOPE intervention consisted of three components: (1) the use of the Fitbit activity tracker, (2) 12-weekly education modules delivered via WeChat, and (3) bi-weekly tailored messages. The 12-week active intervention included the Fitbit tracker and 12 weekly, culturally appropriate, evidence-based educational modules delivered via WeChat, a popular communication app in China. After the 12 weekly modules, the maintenance phase included six bi-weekly tailored tips and messages based on Fitbit data and personal goals sent via WeChat. The control group received general health information without tailoring via WeChat at the same frequency as the SCOPE program. Data on outcomes were collected at baseline, 3 months, and 6 months.

### 2.1. Intervention Contents

**Figure 1:**
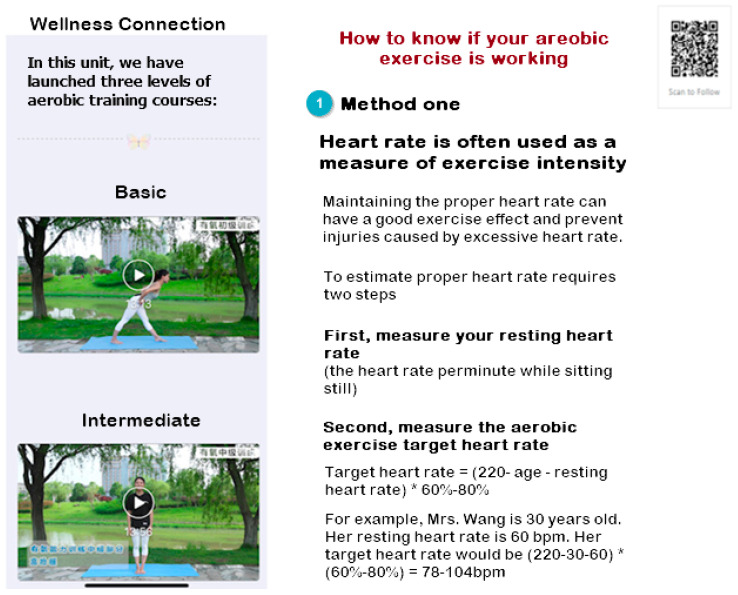
Sample session in WeChat. **Top**: basic aerobic exercise; **Bottom**: Intermediate aerobic exercise.

1. Fitbit tracker. All participants received a Fitbit Alta HR^TM^ tracking device to wear daily. Each participant received in-person, one-on-one training from the research assistants on accessing the app and their tracking data. Fitbit Alta HR^TM^ built by Fitbit (https://www.fitbit.com/gb/shop/altahr (accessed on 1 July 2019)), is a sensitive, 3-D motion sensor that tracks an individual’s activities, sleep, and food intake. The website and apps are currently available in Chinese in China. The Fitbit Alta HR^TM^ has mobile apps (Android and iPhone) that enable real-time automatic wireless syncing to smartphones (via data transfer). Both the Fitbit app and the online program allow tracking of physical activity, sedentary activity, and dietary intake and the ability to set individualized goals, monitor progress toward those goals, and interface with interactive apps related to promoting an active lifestyle, balanced diet, and healthy weight. In the study, participants were asked to wear the Fitbit Alta HR^TM^ daily for at least 10 h while awake and throughout the night while asleep. Those in the intervention group were taught to use the Fitbit and WeChat apps to set up fruit/vegetable intake goals and daily steps. If they did not use the device and app for more than 1 week, a WeChat reminder message was sent to them. Participants in the SCOPE intervention group were asked to share their data with the research team to develop messages tailored to individuals’ behavior patterns.
2. Twelve modules. Modules were sent via WeChat. Participants received 12 weekly, culturally appropriate, evidence-based educational modules along with tailored tips and messages via WeChat. Each module includes an educational session/information that lasts less than 20 min. The educational modules include (1) Assess Your Risk, (2) Prevention Through Cancer Screening and Vaccination, (3) Eat Well to Reduce Cancer Risk for You and Your Family, (4) Smart Shopping and Cooking, (5) Abs Exercise, (6) Active Lifestyle with Your Child to Boost Your Energy, (7) Fitness for You and Your Child, (8) Healthy Lifestyle for Healthy You, (9) Happy Motherhood and Stress Management, (10) Effective Coping and Getting Support, (11) Stay Motivated to Prevent Cancer and Obesity, and (12) Healthy Weight and Flat Abs for Long-Term Health. The contents of the modules were based on relevant evidence from the literature as well as professional organizations in China, such as the Chinese Center for Disease Controls (https://www.chinacdc.cn/en/ (accessed on 1 July 2019)), the National Health Commission of the People’s Republic of China (http://en.nhc.gov.cn/2019-07/17/c_75509.html (accessed on 1 July 2019)), and Beijing Institute for Cancer Research [26]. Please see Figure 1 for a Sample WeChat platform image.
3. Biweekly WeChat messages. Biweekly WeChat messages are based on individual characteristics, behavior patterns, and preferences. Individual characteristics include information related to their health and family environment, such as the number of children at home and family history of breast cancer. Preferences include information collected at baseline on when they would like to receive the WeChat message, and behavior patterns include physical activity and diet data from Fitbit. Text messaging is a convenient way to facilitate realistic goal setting, self-monitoring, and information exchange. Interventions using WeChat-based text messages have been found to promote behavioral change and reduce weight in adults [27,28,29]. Participants received six biweekly WeChat messages to encourage and reinforce positive behavioral changes in this study. The trained research assistant delivered the WeChat messages. The message included tips on lifestyle modification, stress management, and healthy weight maintenance based on users’ characteristics, behavior patterns, and goals. Participants in the intervention group recorded vegetable and fruit intake with specific goals and set up goals for physical activity (steps) in the Fitbit. The data provided the foundation for developing tailored messages based on behavior patterns. For example, the women who had a family history of breast cancer and limited physical activity, but were eating a healthy diet received a tailored message reminding them of the risk of developing cancer, the importance of screening, and supplied exercises that could be performed in 10–15 min at home with or without their children. They also received an encouraging message for reaching their goals. In addition, to increase social support, we encouraged building a social network by sharing healthy recipes and photographs of innovative ideas for improving physical activity and healthy diet with other family members and friends.

### 2.2. Control Group

The control group received a Fitbit Alta HR^TM^ and 12 weekly, non-tailored educational modules via WeChat (˂20 min, the same as the intervention group) on general health topics important to women in China. The topics included anxiety, depression, sexually transmitted infections, intimate partner violence, HIV, unintended pregnancy, hepatitis B, and general cancer prevention. Participants also received six biweekly, non-tailored text messages on general health topics. The 12 modules are different from the modules in the intervention group. These modules in the control group include general and non-tailored information for general health topics. The weekly educational modules sent to the control group are the same duration as for the intervention group to avoid attention bias.

### 2.3. Data Collection and Measures

Data were collected at baseline, 3 months, and 6 months. The research assistants and a registered nurse conducted all measurements. The trained research assistant and registered nurse measured WC, weight, and height. Study participants completed all questionnaires in a separate room where a research assistant could answer questions. All measurements and survey items have been used in China and demonstrated adequate reliability and validity.

1. Feasibility. The number of eligible participants, percentage of completed assessments, and module access were assessed.
2. Usability and acceptance. The frequency of accessing the 12 modules and satisfaction of reviewing the modules were collected to measure the usability and acceptance of these modules.
3. Demographic survey. The socio-demographic questionnaire assesses participants’ age, education level, parents’ occupation, residence, and household income.
4. Waist circumference (WC). This study followed the waist circumference measurement protocol set by the National Institute of Health. Waist circumference was measured midway between the lowest rib and the superior border of the iliac crest. The circumferences were given as the mean of the 2 measurements to the nearest 0.1 cm. Central obesity is a waist circumference ≥ 80.0 cm for Chinese women [30,31,32]. The WC has adequate validity and reliability (r = 0.66–0.89 with % body fat) [31,32].
5. Body mass index BMI. BMI is determined by measuring weight and height (weight [kg]/height [m]^2^). Research assistants measured height and weight using instruments calibrated before each measurement. The instruments have an accuracy of 0.1 cm in height and 0.1 kg in weight. This study used the criteria for overweight and obesity in China criteria: underweight as BMI < 18.5 kg/m^2^, normal weight as 18.5 kg/m^2^ ≤ BMI < 24.0 kg/m^2^, overweight as 24.0 kg/m^2^ ≤ BMI < 28.0 kg/m^2^, and obese as BMI ≥ 28.0 kg/m^2^ [30,31,32].
6. Fruit/vegetable intake and physical activity. Two items measured vegetable and fruit intake (>5 servings/per day) and regular physical activity (a total of 150 min per week) were used in this study. These two items were from the Canadian Diabetes Risk Questionnaire for the Chinese population survey used in China (CHINARISK). Adequate reliability (test-retested reliability = 0.98) and validity (sensitivity = 0.73 and specificity = 0.63) have been reported [33].
7. Physical activity/average daily steps. The average daily steps on the week we collected data were used. Previous studies indicated that the Fitbit step count outputs are stable at different speeds and substantial with observer counts (0.97–1.0) [34]. The reliability of the Fitbit is generally good, with satisfied intraclass correlation coefficients (ICC), Bland-Altmann plots, and limits of agreement (LOAs) results [35]. The Fitbit Alta HRTM has been used in other studies and demonstrated a good level of ICC = 0.76–1.0 and error = 11% [36,37].
8. Self-efficacy for diet and physical activity. The 8-item survey was used in the study. Participants were asked about their confidence level in engaging in activities related to a healthy diet and regular physical activities. Scoring was based on five points (0- not confident to 5- very confident), and a higher score indicates a higher level of self-efficacy. Reliabilities have been documented in the exercise domain (Cronbach’s alpha 0.87 to 0.97) and the diet domain (Cronbach’s alphas 0.93 to 0.95) [38].
9. Social support for diet and physical activity. The social support scale consists of six items measuring social support for diet and physical activity from family members and friends. The survey includes three items to evaluate the support of diet and exercise from family members and three from friends. The diet social support scale is based on a 4-point scale: 0 (never), 1 (sometimes), 2 (frequently), or 3 (always), while the physical activity social support scale is rated on a 5-point scale: 0 (never) to 4 (always). Higher scores indicate more social support for the participant. The survey has adequate reliability, with Cronbach’s alpha ranging from 0.80 to 0.87 for the diet domain and 0.84 to 0.91 for the physical activity domain [39].
10. Perceived stress scale (PSS). This 14-items survey measures the degree of perceived stress the mothers perceived in the past month. The survey asked participants to respond to 14 questions on how often they have felt (such as upset, nervous, angry, or having difficulties) over the past month. Participants report the frequency of these questions on a five-point Likert scale (0  =  never to 4  =  very often), with the higher the score reflecting the higher level of the perceived stress. This survey has been used in China and has good psychometric properties in the Chinese population [40].
11. Breast cancer risk knowledge, attitude, and barriers questionnaire (CBCSBQ). A 12-item self-report questionnaire measures knowledge (4 items), attitude (4 items), and barriers to mammographic screening (4 items) [41,42]. Participants were asked to rate these items on a 5-point Likert scale, with 1 corresponding to the lowest attitude, least knowledge, or greatest barrier. The CBCSBQ has a good Cronbach’s alpha = 0.66–0.72. The goodness of fit index: 0.95–0.98 from previous studies [41,42].

### 2.4. Data Analysis

Because this is a pilot study aimed at testing the feasibility, acceptance, and preliminary efficacy, a power analysis was not conducted. Since this pilot RCT has a limited sample size, analyses were intended to be exploratory, and we set α = 0.05 (two-sided). Descriptive statistics (means and standard deviations, or medians and interquartile ranges if skewed) were reported for quantitative demographic and outcome variables. Frequencies and percentages were reported for categorical variables. T, chi-square, or analysis of variance (ANOVA) tests were used to compare differences between the intervention and control group at baseline. We used mixed effects models to estimate the effect of intervention vs. control from before/after treatment (linear for continuous outcomes; logistic for binary, e.g., yes to 5 servings of fruit and vegetables). Because the sample size was small, we used the nonparametric bootstrap with 5000 draws to obtain bias-corrected confidence intervals and evaluated significance [43,44,45]. Analyses included using intent-to-treat. The standardized effect size for between-group differences in outcomes at 3 and 6 months was estimated using Cohen’s *d*, which has been described as a small effect for 0.2–0.49, a medium effect for 0.5–0.79, and a large effect for ≥0.8 [46]. Data analysis used Stata Statistical Software, Release 17 (StataCorp LLC, College Station, TX, USA).

## 3. Results

### 3.1. Sample Characteristics

**Figure 2:**
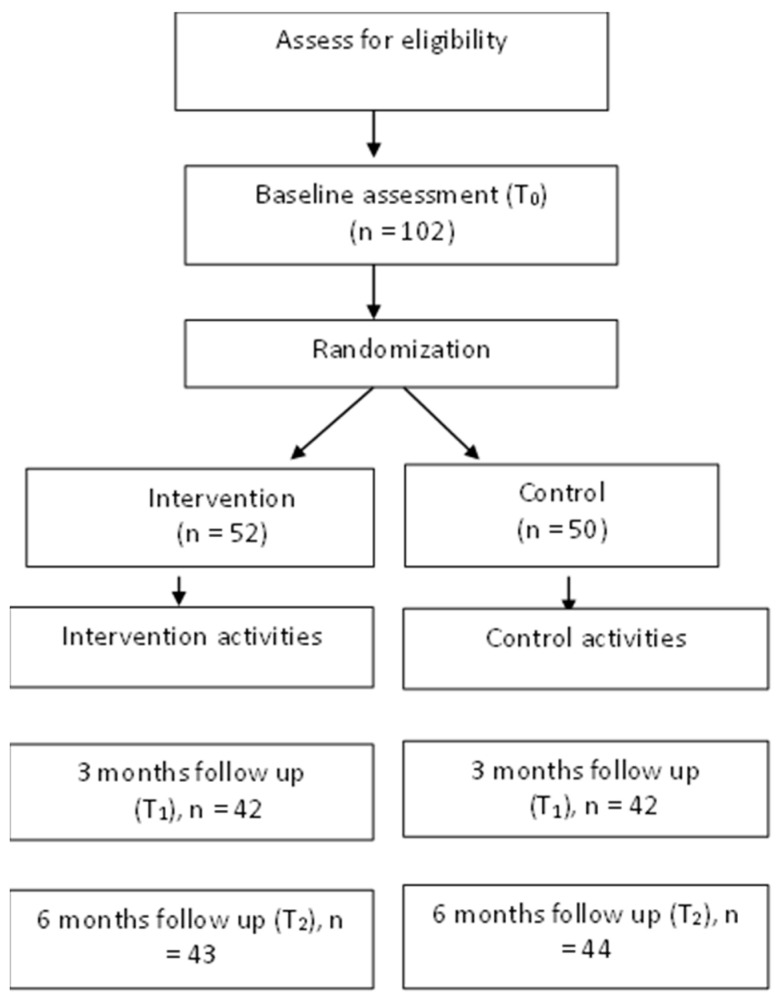
Patient recruitment flow chart.

A total of 102 women (52 in the SCOPE intervention and 50 in the control group) participated in this study (see Figure 2). The mean age was 36.7 years (SD = 0.48), WC was 91.97 cm (SD = 0.89), and BMI was 27.74 (SD = 0.74). A total of 91% of participants were residents of urban areas, and 30% received high school education or less. Most participants work and have a monthly household income greater than 4501 yuan. (The poverty standard is 4000 per person per year in China.) A total of 39.4% of women reported eating five servings of fruit and vegetables, and 43% of women reported exercising daily at the baseline with an average daily step of 10,081 (Table 1 and Table 2). There was no significant difference (*p* > 0.05) in the demographic data and outcome variables at the baseline between the SCOPE and control groups (Table 3).

### 3.2. Effect of the Intervention

Results from the mixed-model analysis found that at the 3-month follow-up, compared to baseline, participants in the SCOPE program compared to control significantly reduced WC (*d* = 0.08, *p* = 0.008) and BMI (*d* = −0.05, *p* = 0.006), and improved daily steps (*d* = 0.63 (a medium effect), *p* = 0.012), positive breast cancer attitude (*d* = 0.81 (large), *p* < 0.001), and breast cancer knowledge (*d* = 0.27 (small), *p* = 0.03; Table 4).

At 6 months, compared to baseline, women in the SCOPE group compared to control significantly reduced WC (*d* = −0.39 (small), *p* < 0.001) and BMI (*d* = −0.18, *p* = 0.001), and increased breast cancer knowledge (*d* = 0.48 (small), *p* = 0.001) and attitude (*d* = 1.39 (large), *p* < 0.01). WC, BMI, and BC attitudes and knowledge were estimated slightly stronger at 6 months than at 3 months, but the step count was slightly less (and no longer significant, *p* = 0.09).

### 3.3. Feasibility and Satisfaction of the SCOPE Modules

A total of 85% of participants completed 6 months of follow-up. The main reasons for missing the follow-up assessment include lack of time and inability to come to the site for assessment. The average number of modules viewed was 8.5 out of 12 modules. Most participants in the SCOPE intervention program (93%) indicated they learned a great deal or a lot from these modules.

## 4. Discussion

As breast cancer is a significant health issue, and obesity increases the risk for breast cancer, especially for women with children, we developed a smartphone-based intervention using a popular communication app tailored to Chinese mothers. Our intervention aimed at reducing obesity, increasing knowledge and attitude, and reducing barriers to breast cancer. In our pilot study, less than 50% of participants who have abdominal obesity reported eating five servings of fruit and vegetables and exercising daily at baseline. We observed a significant reduction in WC and BMI in the intervention group compared to the control group and small to large effect sizes of the intervention on improving PA steps, breast cancer knowledge, and breast cancer attitude at three months follow-up. Smaller effects on behavior and psychosocial outcomes were found at the six-month follow-up. Our pilot study revealed good feasibility and high satisfaction in accessing the modules.

The lower level of engagement in healthy eating and active lifestyle found in women with abdominal obesity in China is problematic and even worse during the COVID pandemic. The results of our study are consistent with other studies. Previous work examining the effects of the COVID pandemic in China found that more than half of people gained weight, with 16.3% of women gaining more than 2.5 kg [47]. They also found that about 38.2% of people increased their snack intake during the lockdown, and 54.3% reported reduced physical activity [48]. Diets contained a lot of soft drinks, fried food, and pickles, with a low intake of fresh vegetables. Furthermore, the rate of inadequate physical activity increased to nearly 70%, which was about five times higher than in the non-pandemic period [47,49]. This is concerning as research indicates that Chinese women have low engagement in healthy behaviors (e.g., exercise, diet) that can increase abdominal obesity and the risk for breast cancer [50,51]. A recent meta-analysis of 36 studies found that low-fat dairy products decrease the risk of breast cancer among premenopausal women (RR = 0.94, 95% CI = 0.89–1.00, *p* = 0.048) [52]. Another meta-analysis review of 32 studies found that prudent dietary patterns reduce breast cancer risk in premenopausal women (RR 0.77, 95% CI 0.61, 0.9) [53]. Women with dependent children have an increased risk of obesity due to stress, insufficient education, and work and family-related demands that prevent their engagement in positive health-related behaviors and early screening activities. Conventional intervention for obesity and breast cancer risk reduction has limited effects, particularly for women who need to manage work, family life, and health. Thus, interventions are critical to addressing the unique stress and lifestyle challenges women with children face.

The results of our study revealed great potential in reducing obesity and the risk of breast cancer. Although studies focusing on Chinese women are limited, current evidence demonstrates that premenopausal Asian women with abdominal obesity have a higher risk of breast cancer compared to non-Hispanic white women [2,54]. The association between unhealthy lifestyle behaviors and obesity is well-known and abdominal obesity is a modifiable risk factor for breast cancer [55,56,57]. The results of our study are consistent with other smartphone intervention studies that have found that obese adults, including both men and women, lost significantly more weight than those in a control group (1.04–6.8 kg) [16,17,19,58,59]. A recent meta-analysis of 20 RCTs from four countries showed that participants in the mHealth program significantly reduced body weight (ranging from −2.84 to −1.87 kg) [60]. Smartphone-based health interventions are viable alternatives to traditional in-person programs. They can be effective for weight management compared to in-person interventions, especially during a pandemic when daily activities, including grocery shopping and outdoor activities, have been limited.

Our study also revealed small to large effects on the behaviors and psychosocial factors, such as increasing fruit/vegetable intake, PA steps, and social support regarding diet and PA. As studies have supported the impact of a poorer health lifestyle, including a sedentary lifestyle and inadequate fruit and vegetable intake, on an increased risk for abdominal obesity, it is important to build a healthy lifestyle and a supportive environment for maintaining a healthy lifestyle to ensure the sustainability of health behavior changes [61,62]. Although this study occurred during the pandemic (2021), the use of communication apps and smartphones keeps the access to health information we developed without interruption. Since the COVID-19 pandemic, digital technology use has grown significantly, increasing by 40% to 100% [63]. The use of digital technology in China for containing COVID and improving social life is booming with government support [64]. In addition, our modules include physical activities that women can do alone and/or with their children and tips for developing/building a supportive environment for themselves and their families.

In addition, this intervention also improved women’s knowledge and attitudes regarding breast cancer. Our study is consistent with other studies suggesting that adequate educational programs can increase women’s knowledge and attitudes about breast cancer [65,66,67]. Studies have suggested that women with adequate knowledge and positive attitudes are more likely to obtain screening and testing [68,69,70]. Having adequate knowledge and a positive attitude toward breast cancer is essential in the early prevention of breast cancer. It may increase early detection and reduce the burden of breast cancer-related treatment.

Our pilot study revealed good feasibility and high satisfaction in accessing the modules. Traditional in-person weight management and breast cancer prevention programs are time-consuming and costly, especially during a pandemic. The promising effect of our intervention can contribute to the use of communication apps and theoretical-based intervention. Our intervention integrates the SCT and SDH, the use of everyday technology (WeChat), and an adaptation of an evidence-based program tailored to the behavior patterns, preferences, and cultures of women in China. WeChat is a popular multifunctional social media mobile app, and connectivity is widely available without restrictions [71]. Using WeChat as an education platform is successful and feasible for promoting weight loss in obese adults, including women in China [72,73], and controlling excessive weight gain in pregnant women [74]. Results of our study suggest that implementing smartphone technologies for weight management and breast cancer prevention may have great potential for decreasing the obesity and breast cancer epidemic while simultaneously improving the health behavior of women who have abdominal obesity.

There are several limitations in this pilot study. The study’s results need to be interpreted with caution. Because this pilot study was conducted with a small sample size and a homogeneous population, we have limited power to detect small differences. The results may also only be generalized to similar populations. Moreover, this study did not include other potential risk factors such as alcohol drinking and smoking. Other limitations included convenience sampling, self-reported measures, and only 6 months of follow-up. Future studies would also need to investigate whether specific subgroups of women can benefit more from this program and include other potential risk factors (such as alcohol consumption and smoking) in the study.

## 5. Conclusions

This pilot study demonstrates a promising way to prevent obesity and breast cancer. The SCOPE intervention delivered via a communication app is feasible and may positively reduce women’s risk for obesity and improve breast cancer knowledge and attitude. This intervention has great potential to promote the health and wellness of women and the community.

## Figures and Tables

**Table 1:** Demographics.

	Total (*n* = 102)		Control (*n* = 50)		Experimental (*n* = 52)		t/χ^2^	*p*
Age, mean (SD)	36.76	(4.84)	37.4	5.43	369	4.14	1.40	0.16
BMI, mean (SD)	27.785	(3.70)	27.68	3.46	27.89	3.94	−0.46	0.64
Ethnicity (%)							0.53	0.47
Han Chinese	92	(90.2)	44	(88)	48	(92.31)		
Minority	10	(9.8)	6	(12)	4	(7.69)		
Residence (%)							821.00	0.77
Urban	91	(91)	45	(91.84)	46	(90.2)		
Rural	9	(9)	4	(8.16)	5	(9.8)		
Education (%)							0.21	1.00
Middle school	4	(3.92)	2	(4)	2	(3.85)		
High school	27	(26.47)	13	(26)	14	(26.92)		
Some college	21	(20.59)	10	(20)	11	(21.15)		
Bachelor’s degree	43	(42.16)	22	(44)	21	(40.38)		
Graduated school	7	(6.86)	3	(6)	4	(7.69)		
Occupation (%)							8.39	0.21
Workers	5	(4.9)	3	(6)	2	(3.85)		
Civil servant	19	(18.63)	12	(24)	7	(13.46)		
Self-employed	22	(21.57)	8	(16)	14	(26.92)		
Teachers	10	(9.8)	7	(14)	3	(5.77)		
Healthcare Practitioners	14	(13.73)	5	(10)	9	(17.31)		
Unemployed	2	(1.96)	2	(4)	0	(0)		
Others	30	(29.41)	13	(26)	17	(32.69)		
Household income (%)							4.54	0.34
401–1500 RMB	1	(0.98)	0	(0)	1	(1.92)		
1501–4500 RMB	11	(10.78)	3	(6)	8	(15.38)		
4501–9000 RMB	29	(28.43)	15	(30)	14	(26.92)		
>9000 RMB	60	(58.82)	32	(64)	28	(53.85)		
None	1	(0.98)	0	(0)	1	(1.92)		

**Table 2:** Baseline data.

Variable	All	SCOPE *n* = 52	Control *n* = 50	*p*
Age (years)	36.76 (4.84)	36.1 (4.14)	37.45 (5.43)	0.08
WC (cm)	91.97 (8.95)	96.61 (9.79)	91.3 (8.04)	0.76
BMI	27.74 (3.65)	27.95 (3.84)	27.52 (3.46)	0.72
Diet 5 F/V Yes	40/101	17/51	23/50	0.19
PA (steps)	10,081 (4672)	10,480 (4695)	9494 (4646)	0.83
Diet SE	6.9 (3.39)	7.14 (2.97)	6.65 (3.78)	0.76
PA SE	7.49 (3.64)	7.38 (3.63)	7.6 (3.69)	0.38
Diet SS	11.22 (1.86)	11.31 (1.69)	11.12 (2.04)	0.69
PA SS	5.98 (2.63)	5.59 (2.51)	6.38 (2.71)	0.07
PSS	28.2 (6.18)	28.13 (6.72)	28.27 (5.6)	0.46
BC Attitude	0.42 (0.17)	0.40 (0.18)	0.43 (0.16)	0.15
BC Knowledge	0.54 (0.19)	0.52 (0.21)	0.55 (0.16)	0.17
BC barrier	0.52(0.16)	0.55 (0.16)	0.52 (0.15)	0.79

**Table 3:** Comparisons between control and SCOPE experimental groups.

	Control		Experimental				
	obs	Mean	obs	Mean	St Err	T	*p*
T0 WC	50	91.30	52	92.61	1.78	−0.75	0.47
T1 WC	42	90.37	38	90.92	1.99	−0.30	0.78
T2 WC	44	91.04	43	88.82	1.81	1.25	0.22
T0 BMI	50	27.52	52	27.95	0.73	−0.60	0.55
T1 BMI	42	27.76	38	28.17	0.85	−0.50	0.63
T2 BMI	44	27.79	42	27.57	0.81	0.30	0.78
T0 PSS	48	28.27	52	28.13	1.24	0.10	0.91
T1 PSS	41	28.15	40	26.93	1.30	0.95	0.35
T2 PSS	43	27.88	43	27.07	1.29	0.65	0.53
T0 Diet self-efficacy	49	6.65	51	7.14	0.68	−0.70	0.48
T1 Diet self-efficacy	42	6.93	40	7.03	0.64	−0.15	0.88
T2 Diet self-efficacy	44	6.84	43	6.49	0.64	0.55	0.58
T0 PA self-efficacy	50	7.60	52	7.39	0.72	0.30	0.77
T1 PA self-efficacy	42	6.45	40	6.58	0.72	−0.15	0.87
T2 PA self-efficacy	44	6.82	43	6.70	0.67	0.20	0.86
T0 Diet social support	50	11.12	51	11.31	0.37	−0.50	0.60
T1 Diet social support	41	11.73	40	11.45	0.46	0.60	0.54
T2 Diet social support	43	10.72	42	11.43	0.42	−1.65	0.10
T0 PA social support	50	12.82	52	11.27	0.81	1.90	0.06
T1 PA social support	42	11.45	40	11.70	0.80	−0.30	0.76
T2 PA social support	43	12.42	43	11.23	0.87	1.35	0.18
T0 Step	34	9494.82	50	10,480.27	1039.37	−0.95	0.35
T1 Step	23	7013.33	32	10,989.04	1389.44	−2.85	0.01
T2 Step	25	4306.83	31	7756.10	1574.04	−2.20	0.03
T0 Attitude	50	91.30	52	92.61	1.78	−0.75	0.47
T1 Attitude	42	90.37	38	90.92	1.99	−0.30	0.78
T2 Attitude	44	91.04	43	88.82	1.81	1.25	0.22
T0 Knowledge	50	27.52	52	27.95	0.73	−0.60	0.55
T1 Knowledge	42	27.76	38	28.17	0.85	−0.50	0.63
T2 Knowledge	44	27.79	42	27.57	0.81	0.30	0.78
T0 Barrier	48	28.27	52	28.13	1.24	0.10	0.91
T1 Barrier	41	28.15	40	26.93	1.30	0.95	0.35
T2 Barrier	43	27.88	43	27.07	1.29	0.65	0.53
Veg/Fruit T0 (yes)	44	21 (47.7%)	43	21 (48.8)		0.01	0.92
Veg/Fruit T0	50	23 (46%)	51	17 (33.3%)		1.7	0.19
Veg/Fruit T0	42	17 (40.5%)	40	22 (55%)		0.73	0.19

**Table 4:** Mixed model and effect size of the intervention.

Outcome Variable	Coeff (SE) or OR	Z (*p*)	95% CI	Standardized Effect Size
WC (cm)3 months	−2.31 (0.88)	−2.64 (0.008)	−4.03, −0.59	−0.08
WC (cm) 6 months	−4.40 (0.85)	−5.19 (<0.001)	−6.06, −2.74	−0.39
BMI 3 month	−0.65 (0.23)	−2.77 (0.006)	−1.11, −0.19	−0.05
BMI 6 month	−0.87 (0.23)	−3.82 (<0.001)	−1.32, −0.42	−0.18
Diet (5 F/V Yes) 3 month	OR = 3.06 (1.86)	1.84 (0.06)	0.93, 10.06	0.62
Diet (5 F/V Yes) 6 month	OR = 1.78 (1.06)	0.97 (0.33)	0.56, 5.71	0.32
PA (steps) 3 month	3101 (1231)	2.52 (0.012)	685.4, 5517.0	0.63
PA (steps) 6 month	2076 (1223)	1.7 (0.09)	−321.2, 4473.5	0.52
Diet SE 3 month	−0.19 (0.64)	−0.30 (0.77)	−1.44, 1.07	0.11
Diet SE 6 month	−0.61 (0.63)	−0.97 (0.33)	−1.84, 0.62	0.18
PA SE 3 month	0.15 (0.78)	0.19 (0.85)	−1.38, 1.68	0.10
PA SE 6 month	−0.04 (0.77)	−0.05 (0.96)	−1.54, 1.47	0.03
Diet SS 3 month	−0.47 (0.54)	−0.88 (0.38)	−1.52, 0.58	0.25
Diet SS 6 month	0.53 (0.53)	0.99 (0.32)	−0.51, 1.56	0.28
PA SS 3 month	1.52 (0.79)	1.92 (0.055)	−0.03, 3.08	0.51
PA SS 6 month	0.39 (0.78)	0.50 (0.62)	−1.14, 1.92	0.12
PSS 3 month	−1.1 (1.19)	−0.94 (0.35)	−3.44, 1.21	−0.17
PSS 6 month	−1.12 (1.16)	−1.02 (0.31)	−3.47, 1.09	−0.10
BC Attitude 3 month	0.14 (0.03)	4.77 (<0.001)	0.08, 0.19	0.81
BC Attitude 6 month	0.17 (0.03)	6.11 (<0.001)	0.12, 0.23	1.39
BC Knowledge 3 month	−0.02 (0.02)	2.22 (0.03)	0.008, 0.13	0.27
BC Knowledge 6 month	0.01 (0.02)	3.23 (0.001)	0.04, 0.16	0.48
BC barrier 3 month	0.03 (0.03)	1.06 (0.29)	−0.02, 0.09	0.19
BC barrier 6 month	0.04 (0.03)	1.57 (0.11)	−0.02, 0.10	0.26

## Data Availability

The data presented in this study are available on request from the corresponding author. The data are not publicly available due to them containing personal information.
